# Micropropagation Protocol and Genetic Stability of the *Salix myrtilloides* Plants Cultivated In Vitro

**DOI:** 10.3390/biology12020168

**Published:** 2023-01-20

**Authors:** Marzena Parzymies, Magdalena Pogorzelec, Katarzyna Głębocka, Elwira Sliwinska

**Affiliations:** 1 Institute of Horticultural Production, University of Life Sciences in Lublin, 20-612 Lublin, Poland; 2 Department of Hydrobiology and Protection of Ecosystems, University of Life Sciences in Lublin, 20-262 Lublin, Poland; 3 Institute of Plant Genetics, Breeding and Biotechnology, University of Life Sciences in Lublin, 20-950 Lublin, Poland; 4 Laboratory of Molecular Biology and Cytometry, Bydgoszcz University of Science and Technology, 85-796 Bydgoszcz, Poland

**Keywords:** acclimatization, active conservation, cytokinins, flow cytometry, ISSR, multiplication rate, somaclonal variation, tissue culture

## Abstract

**Simple Summary:**

*Salix myrtilloides* is a relict species endangered with extinction in many Central European countries. To save the populations on the southern border of its range, in vitro propagation was used for plants production because it allows one to obtain a lot of new plants in a relatively short time without damaging the existing ones. We collected shoot pieces and multiplied them in a growing media, which contained nutrients and growth regulators. When we produced enough daughter plants, we planted them into soil and hardened them to natural conditions. Based on the conducted genetic analysis and flow cytometry, we stated that obtained plants are genetically identical to the mother ones. The conducted research confirmed that tissue culture may be used to propagate the endangered *S. myrtilloides* species and that the obtained plants may be used to establish new populations or to strengthen the existing ones.

**Abstract:**

*Salix myrtilloides* L. is a relict species, threatened with extinction in many European countries. To prevent the loss of the species, tissue culture was established to produce plant material for reintroduction in natural habitats. Micropropagation was chosen as a method to obtain new plants. *S. myrtilloides* shoots were disinfected with NaOCl, AgNO_3_, or with a two-step disinfection with NaOCl, and then placed on MS medium supplemented with BA at 1 mg·dm^−3^ and IBA at 0.1 mg·dm^−3^. Regenerated shoots were cultivated in presence of BA, KIN, and 2iP to select the treatment with the highest multiplication rate. The obtained plants were acclimatized to ex vitro conditions. Inter-simple sequence repeat (ISSR) and flow cytometric analyses were conducted on in vitro regenerated plants to check their genetic stability. The best disinfection results were obtained when explants were treated with 1.5% NaOCl for 20 min. The highest multiplication rate and good quality plants were noted in the control media, without growth regualtors and in presence of kinetin at 0.5 mg·dm^−3^. Flow cytometry and ISSR analyses confirmed genetic stability in plantlets, which indicated the possibility to use the in vitro obtained plants for reintroduction.

## 1. Introduction

In the 21st century, the conservation of biodiversity is one of the most important challenges for both nature conservation services and scientists looking for effective methods of preventing extinction and supporting the survival of endangered species [1,2]. 

Preserving the population resources of endemic or relict plant species, especially those of boreal origin, at the southern border of their geographical range is currently one of the challenges dependent on global climate change [3,4].

In the case of the rarest species of plants and animals, the form of active protection usually recommended is reintroduction. It is a form of species translocation, which aims to return the population to the site where it functioned before or to strengthen the existing populations with specimens from ex situ cultivation [5,6,7].

The importance of ex situ conservation is included in Article 9 of the Convention on Biological Diversity (CBD) and Target 8 of the Global Strategy for Plant Conservation, which refer to ex situ and in situ conservation of rare and vulnerable species [8] and indicates that there is a need to integrate both types of methods to optimize the effects [9].

In the case of plants, it is a combination of reintroduction and in vitro propagation, which are currently considered as the most effective methods for preserving the genetic resources of species threatened by extinction. Micro-reproduction allows one to obtain new, healthy plants that origin from the natural resources of the species, even if they are already on the verge of dying out [10,11]. According to Sheikholeslami et al. [12], plants obtained in vitro and introduced into the natural environment are generally in good condition. Kułak et al. [13] reported that in vitro cultivation may be used in protection and preservation, sustainable management, restoration, and rewilding.

Ex situ conservation with the use of biotechnological techniques requires one to establish methods for explants disinfection, tissue culture initiation, multiplication, rooting, acclimatization of the plant species, and evaluation of its genetic stability [14]. Plant tissue culture is typically initiated from the plant material collected in the field, which must be disinfected, as contaminations are the main problem during the initiation of tissue culture [15]. Somaclonal variation is also a common problem when cultures are cultivated for a long time or in presence of growth regulators [16,17,18,19,20,21]. The confirmation of the genetic true-to-typeness of in vitro propagated plants is recommended, especially before reintroduction into natural stands [22,23,24,25,26]. Molecular markers, such as random amplified polymorphic DNA (RAPD), inter-simple sequence repeat (ISSR), or amplified fragment length polymorphism (AFLP) might be used as a way to detect genetic variations [27,28,29]. Flow cytometry, applied for genome size assessment, is commonly used to complement molecular marker analyses [23,30]. On the other hand, the proper genetic differentiation is important for survival of a population, as it allows adaptive variation in changing conditions [31].

In vitro techniques were used to propagate a large number of plants threatened with extinction, but there are no data on the micropropagation of *Salix myrtilloides*.

*S. myrtilloides* L. (swamp willow) is an endangered relict species in many Central European countries. It occurs in isolated locations, in the vicinity of the southern and western limits of its geographical range. Populations of the species decline continuously, but there are very few reports concerning their active protection [32].

*S. myrtilloides* is a low-growing shrub, with small, bluish-green leaves covered with wax. Branches and shoots are thin and erect, initially glabrous, and yellowish brown or red, while older ones are grey or reddish brown.

The literature data indicate that the species reproduces mainly vegetatively in natural conditions, but the results of research conducted in Poland indicate that the populations of this species are highly genetically diverse and do not consist of clones. The possibility of the generative reproduction was confirmed by the data on the high viability of gametophytes and seeds in the studied populations [33,34].

*S. myrtilloides* is a photophilous species. It occurs in flooded and non-wooded, lowland, and submontane transitional bogs, and it prefers acidic peat with pH ranging from 3.5 to 5.5 [35,36,37].

It is a species native to subarctic regions of northeastern Europe and northern Asia, to the Pacific Ocean coasts in the east, with isolated populations further south in mountain bogs in the Alps, Carpathians, and Sichote-Aliń mountains. Its distribution in Central Europe is highly fragmented. In the Czech Republic, Slovakia and Poland, the species is considered to be a glacial relict, critically endangered, and currently known only from a few sites [38,39,40]. In the 1950s, it was present in about 90 stands in Poland, but most of them disappeared. The results of the monitoring confirmed the reduction of population resources by about 80% [32].

*S. myrtilloides* has been legally protected in Poland since 1983, and it is included in the red list of plants and fungi in Poland as a critically endangered species (category of threat—E) and in the Polish red data book of plants as an endangered species (category of threat—EN) [39]. The main threats to populations of *S. myrtilloides* are indirect or direct human activities, habitat eutrophication, drainage of peat bogs, and expansion of other species [41,42].

*S. myrtilloides* requires active conservation in natural stands and the protection of its genetic resources. We describe the methods of in vitro cultures using plant material from the last remaining populations of this species in eastern Poland. In vitro propagation seems the only effective method, as generative propagation via seeds was not successful, and there are not enough donor plants to use other methods of vegetative production. The ISSR and flow cytometry analyses were performed to assess the genetic stability of micropropagated material.

## 2. Materials and Methods

### 2.1. Tissue Culture Initiation and Stabilization

We collected the shoot fragments of *Salix myrtilloides* (L.) from the biggest population in eastern Poland, located in mid-forest peat bog Dekowina (N51°26′39.78″; E23°31′08.94″), Sobibór Landscape Park, in April–May. We excised the plant material according to the permit issued by the Regional Director for Environmental Protection in Lublin (Permit no. WPN.6400.1.4.2014.JR).

In the laboratory, we disinfected the pieces. At the beginning, we shook them three times for 20 min. in tap water with a drop of detergent (Ludwik, GRUPA INCO S.A., Góra Kalwaria, Poland), then we soaked the explants in the 2 mL dm^−3^ water solution of Amistar 250 SC fungicide (azoxystrobin 25 g dm^−3^) (Syngenta, Basel, Switzerland). As a next step, we dipped the pieces in 70% alcohol for 10 s. At the end, we disinfected the explants with 1% NaOCl (sodium hypochlorite) (Chempur, Piekary Śląskie, Poland) for 20 min. We rinsed disinfected explants three times in a sterile distilled water.

Because of a high contamination rate, we also treated plant material with NaOCl at higher 1.5% concentration or with 0.5% water solution of AgNO_3_ (Sigma-Aldrich, Saint Louis, MO, USA) for 15 min. We also applied a two-day disinfection, which was used in the case of *Salix lapponum* [43]. On the first day, we soaked explants in 0.5% NaOCl and placed on MS medium, and the next day we again soaked them in 0.5% NaOCl for 5 min, after which we rinsed explants in distilled water.

We cultivated explants on a Murashige and Skoog (MS) medium [44], with the addition of growth regulators: 1 mg dm^−3^ BA (benzyladenine) (Sigma-Aldrich, Saint Louis, MO, USA) and 0.1 mg dm^−3^ IBA (indolebutyric acid) (Sigma-Aldrich, Saint Louis, MO, USA). We set pH to 5.5. We added 6.75 g dm^−3^ of Microbiological Lab-Agar (BioMaxima S.A., Lublin, Poland) to solidify the media before autoclaving (121 °C and 1 hPa for 21 min).

After disinfection, we cut shoots into pieces with one or two nodes and inserted them into tubes, with one explant per tube. We placed tubes in a growing room at 22± °C, 16-h photoperiod and 35 µmol·m^−2^·s^−1^ light intensity. After 3 cycles, we cultivated the free-from-contamination shoots in 500 mL jars. We subcultured the regenerating shoots every 6 weeks until we obtained enough explants to conduct further studies.

### 2.2. Multiplication

To determine the optimal multiplication media composition, we placed two-nodal shoot tip pieces on MS medium with the addition of: BA, KIN (kinetin), or 2iP (isopentenyl-adenine) (Sigma-Aldrich, Saint Louis, MO, USA), at concentrations of 0.1, 0.5, 1, or 2.5 mg·dm^−3^.

After 5 weeks, we counted the shoots, axillary shoots and roots, measured and weighed them, and calculated the multiplication rate (number of nodes + number of axillary shoots/% of shoots with axillary shoots).

### 2.3. Rooting and Acclimatization

We noticed that shoots rooted spontaneously, and we decided to use them for acclimatization. We took rooted shoots out from jars and washed them under tap water. We cut the roots to 1.5 cm, if needed. We inserted 10 plants into 1 dm^3^ containers filled with a mixture of two types of peat (pH 3.5–4.5 and 5.5–6.5, respectively), sand, and perlite (1:1:1:1 *v*/*v*). We placed containers in glass tanks and covered them with plastic foil to keep high humidity. We maintained the temperature and photoperiod conditions at the same level. After two weeks, we started to harden the plants, removing the foil gradually. Four weeks later, we transferred plants into P9 pots (9 × 9 × 9 cm) containing the same mixture of soil and, additionally, Azofoska universal fertilizer (Grupa INCO S.A., Susz, Poland) (NPK (MgO + SO_3_) = 13:6:17 (4.5 + 21) + microelements) at a concentration of 1 g·dm^−3^. When plants achieved height of 25 cm, we cut them to about 10 cm to stimulate branching. After about 5–6 months, we obtained plants ready for reintroduction into natural stands.

### 2.4. ISSR Analysis

We isolated DNA from 2–3 leaves from 31 regenerants from in vitro cultivation and from a mother plant growing in a natural habitat. We implemented the procedure described by Porebsky et al. [45], with minor modifications. We diluted each sample to 20 ng/µL after measuring DNA concentration and purity with a Nanodrop ND-1000 spectrophotometer (Thermo Fisher Scientific, Waltham, MA, USA). We performed ISSR analysis in 10 µL solution. Composition and reagents concentrations are presented in Table 1, while the thermocycling program is presented in Table 2.

We performed electrophoresis on ethidium bromide stained agarose gels, and we determined product size comparing to NzyDNA Ladder VII (NZYTech, Lda.—Genes & Enzymes, Lisbon, Portugal). We used Past software ver. 4.09 [46] to compute Dice similarity indexes.

### 2.5. Flow Cytometry

Flow cytometric analyses were performed on leaves of in vitro regenerated plants. Samples were prepared, as previously described [22], using Galbraith’s nuclear isolation buffer [47] supplemented with 2% (*w*/*v*) polyvinylpyrrolidone (PVP-10), propidium iodide (PI; 50 µg/mL), and ribonuclease A (50 µg/mL). *Solanum lycopersicum* cv Stupicke (2C–1.96 pg) [48] was used as an internal standard. For each sample, PI fluorescence was measured in at least 5000 nuclei using a CyFlow Ploidy Analyzer flow cytometer (Sysmex Partex, Kobe, Japan). Histograms were analyzed using CyView 1.6 software. The coefficient of variation (CV) of the G_0_/G_1_ peak of *Salix* ranged between 3.77 and 6.86%. Nuclear DNA content was calculated based on the linear relationship between the ratios of the 2C peak positions of *Salix*/*Solanum* on a histogram of fluorescence intensities. Genome size was estimated for five control plants (leaves collected in natural habitat) and 300 in vitro-derived plants.

### 2.6. Statistical Analysis

Statistical analysis of the data was conducted by analysis of variance for orthogonal and non-orthogonal single-factorial design using ARSTAT software. The significance of differences between the means was determined with Tukey’s test at the significance level of *p* = 0.05.

GenAlex 6.51b2 software was used to determine the proportion of polymorphic bands in each group of clones [49,50].

## 3. Results and Discussion

### 3.1. Tissue Culture Initiation and Stabilization

**Figure 1:**
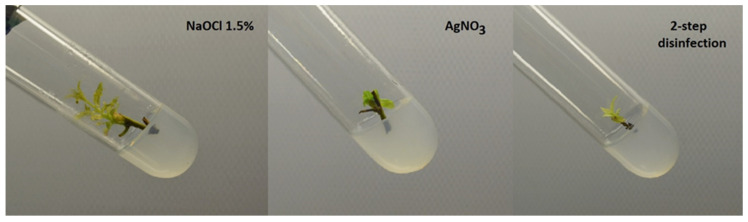
Shoots of *Salix myrtilloides* disinfected with NaOCl, AgNO_3_, or 2-step disinfection after two weeks of cultivation in vitro.

To obtain free of contamination and regenerating explants, the plant material was disinfected with 1.5% NaOCl or with 0.5% AgNO_3_ or with a two-day disinfection method. The use of NaOCl at a concentration of 1.5% for 30 min allowed one to obtain the highest number of regenerating shoots without symptoms of contaminations (39%) (Table 3). The lowest number of regenerating contamination-free shoots was noted in the case of AgNO_3_ application (14%). That solution caused occurrence of the highest number of necrotic explants (72%). The regenerating and free of contamination representative shoot from each treatment is presented in Figure 1.

Initiation of free of contamination tissue cultures of ligneous plants with high morphogenetic activity, such as willows, is one of the most difficult stages of in vitro cultivation [51]. Additionally, the regenerative ability of explants is often reduced after disinfection. In the present research, the number of free of contamination explants was rather low (maximum 39%), which might result from the conditions in which *S. myrtilloides* is growing, peat bogs with high humidity, which enhances the growth of fungi. A similar, high level of contaminations was observed in the case of *Salix lapponum*, growing in similar habitats. Skalová et al. [43] obtained the highest number of contamination-free regenerating shoots of *S. lapponum* when the two-step disinfection method was applied, while in the study of Parzymies et al. [52], it was mercuric chloride, which was not tested in the present experiment. All the regenerating contamination free explants were used to multiply shoots for further studies.

### 3.2. Multiplication

**Figure 2:**
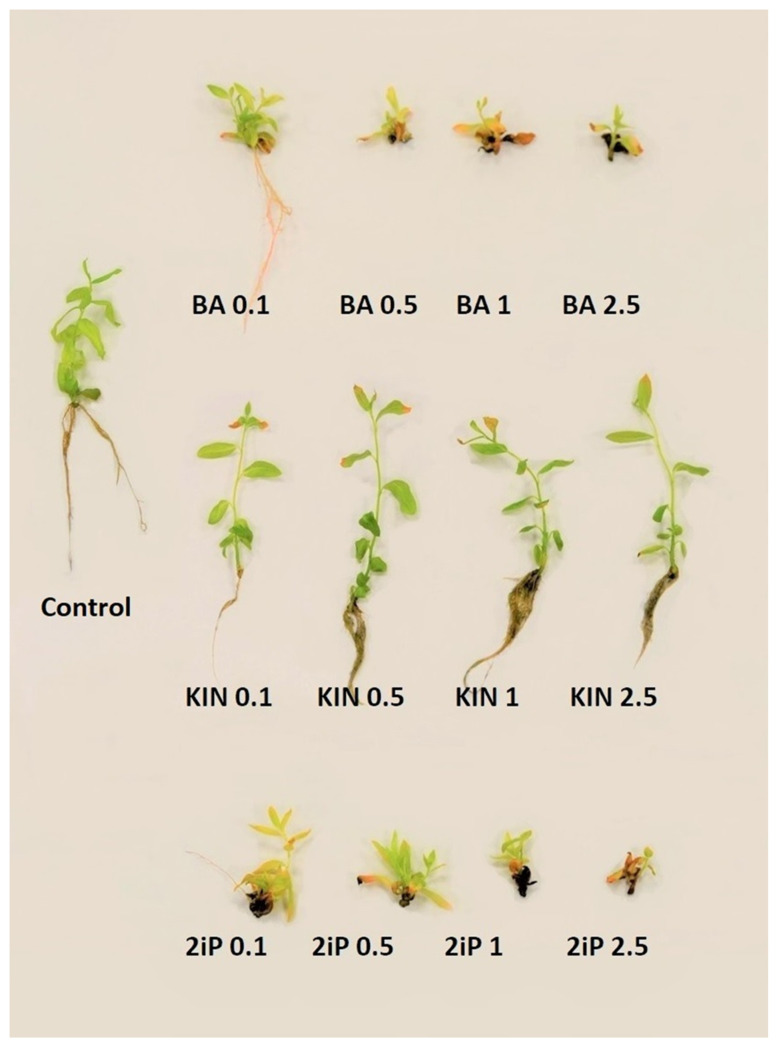
The representative *S. myrtilloides* shoots cultivated in vitro in the media supplemented with different cytokinins.

The composition of the culture media influenced the development, multiplication rate, and rooting of shoots (Table 4 and Table 5, Figure 2). *S. myrtilloides* cultivated in tissue culture grew upright. They were cut into smaller one- or two-nodal pieces, which were placed on the media and grew and produced axillary shoots, which were used for further propagation.

The highest shoots were formed in the media supplemented with KIN at a concentration of 0.5 or 1 mg·dm^−3^ (67.26 and 68.05 mm, respectively) (Table 4). A similar length of shoots was obtained in presence of KIN 0.1 or 2.5 mg·dm^−3^, and BA 0.5 mg·dm^−3^. Regenerating shoots formed the highest number of nodes in the control media (11.26) and the media supplemented with BA 0.5 mg·dm^−3^ (10.90). A statistically, similar number was obtained in presence of BA at concentrations of 1 and 2.5 mg·dm^−3^ (8.50 and 6.35), as well as KIN at concentrations of 0.5 and 1 mg·dm^−3^ (9.21 and 8.42, respectively). The lowest number of nodes was obtained in the media with 1 mg·dm^−3^ of 2iP.

Regenerating shoots formed axillary shoots (Table 4). The highest number of branching shoots was observed in the media with 2iP 0.5 mg·dm^−3^ (100%) and BA 1 mg·dm^−3^ (90%). A high number of shoots with axillary shoots was also noted in presence of BA 0.1 and 0.5 mg·dm^−3^ (67 and 65%, respectively) and 2iP 0.1 and 1 mg·dm^−3^ (79 and 69%, respectively).

Based on the number of nodes on the main shoot, the percentage of branching shoots, and the number of axillary shoots, the multiplication rate was calculated (Table 4). The highest multiplication rate (Mn) was obtained in the media supplemented with 2iP at concentrations of 0.1 and 0.5 mg·dm^−3^ (7.41 and 6.77, respectively). A high multiplication rate was also obtained in presence of 0.5 and 1 mg·dm^−3^ BA (6.80 and 6, respectively). However, based on visual observations, shoots cultivated on those media were deformed and brownish (Figure 2). Shoots growing in the control media and in presence of KIN at all concentrations were characterized by much better quality, and they were long and green. The highest multiplication rate among those treatments was obtained in the control media, 5.78, and in the presence of KIN 0.5 mg·dm^−3^–4.77 (Table 4).

In the present research, the multiplication rate depended mainly on the number of nodes that could be used for further propagation of shoots, which is the common method of in vitro cultivation of willows [53]. The propagation rate of plants in tissue culture depends very often on the use of cytokinins. They are essential during in vitro multiplication, but their type and concentration are different for each species. In the case of willow species, the most often used cytokinins are KIN and BA. Grendysz et al. [54] used kinetin in different concentrations during multiplication of the three varieties of *S. viminalis* and proved that the most advantageous was the medium supplemented with 0.5 mg·dm^−3^ KIN. In the case of *S. lapponum*, KIN at a concentration of 0.5 mg·dm^−3^ and IAA at 0.05 mg·dm^−3^ were used during the multiplication stage [52]. That cytokinin had an advantageous effect in the present research as well. Skalová et al. [43] obtained a high multiplication rate for *S. lapponum* in tissue culture in MS medium containing BA at a concentration of 0.1 mg·dm^−3^ and IBA at 0.01 mg·dm^−3^. Brandova et al. [55] also used MS medium supplemented with 0.1 mg·dm^−3^ BA and 0.01 mg·dm^−3^ IBA for in vitro propagation of a downy willow. A high multiplication rate in the presence of that growth regulator was noted also in the present study, but the plants were characterized with much worse quality than in presence of kinetin or in the control medium.

### 3.3. Rooting and Acclimatization

In the present study, *S. myrtilloides* shoots rooted spontaneously in tissue culture supplemented with cytokinins or in the control media (Table 5). The percentage of rooted shoots and the number of roots depended on the growth regulators used.

All plants produced roots in the control medium or when the media were supplemented with BA at concentrations of 0.5 and 1 mg·dm^−3^, as well as KIN at 0.1 and 0.5 mg·dm^−3^. Shoots did not produce roots in the presence of 2iP at concentrations of 0.5–2.5 mg·dm^−3^ (Table 5). In the media with BA 1 and 2.5 mg·dm^−3^, or KIN at 0.1 and 0.5 mg·dm^−3^, there were more roots per shoot (5.45, 5.13, 5.31) than in presence of BA at 0.1 mg·dm^−3^ (2.00).

Spontaneous rooting is observed in *Salix* sp. cultivated in vitro in the media supplemented with cytokinins alone or in combination with auxins. Skalová et al. [43] reported that *S. lapponum* produced the highest number of roots in presence of BA 0.1 mg·dm^−3^ and IBA 0.01 mg·dm^−3^. In the research of Grendysz et al. [54], kinetin in different concentrations did not influence the rooting of *S. viminalis* in vitro, however, the addition of 0.5 mg·dm^−3^ of KIN was the most effective. In the case of *S. lapponum*, shoots formed roots in the presence of KIN at 0.5 and 1 mg·dm^−3^ and 2iP at 0.5 mg·dm^−3^ [52]. In the present study, both cytokinins, BA and KIN at low concentrations, advantageously influenced the rooting of shoots.

**Figure 3:**
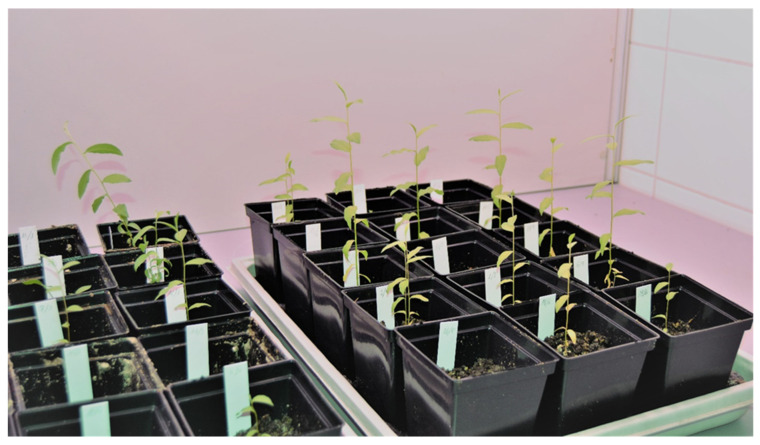
Plants obtained after six weeks of acclimatization after transplanting into P9 pots.

Shoots cultivated in the control medium and in the medium supplemented with KIN 0.5 mg·dm^−3^ were used for acclimatization. After four weeks 70% of plants survived. The plants obtained after six weeks of acclimatization are presented in Figure 3.

### 3.4. ISSR Analysis

**Figure 4:**
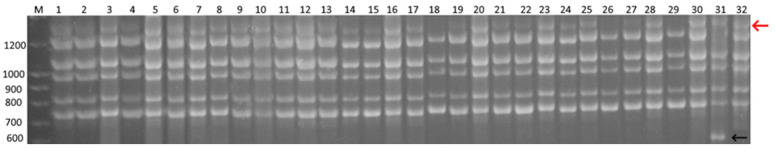
PCR products of SR75 primer. Description of lanes: M—marker, 1–30 regenerated plants, 31—regenerated plant that was excluded from analysis, 32—mother plant. The red arrow shows polymorphic product and the black one shows the band that was amplificated only in excluded plant.

DNA was isolated from 31 regenerated plants and a mother plant. Five ISSR primers were selected out of 15 initially surveyed (Table 6). Altogether, 34 PCR products were amplified. One regenerated plant phenotypically greatly varied from the others, and this difference was also reflected in ISSR analysis. There were three PCR products that were present only in this plant and four that were amplificated in every regenerated plant but not in this one. As it was obvious that this plant cannot be put into the environment, it was excluded from the rest of the analysis. So, finally, 31 products were obtained, out of which six were polymorphic (Figure 4). The values of all Dice similarity indexes were above 0.9.

Agarose gels depicting molecular marker analysis of in vitro regenerated and a mother plant should perform only monomorphic bands, which indicates that no somaclonal variation occurred during micropropagation, especially when that process did not cover callus formation [56]. However, in the case of *S. myrtilloides*, six polymorphic bands out of 31 altogether amplified were obtained, which corresponded to 19.4%. Those plants did not differ morphologically from each other, and the general growth habit was typical for the species. Godwin et al. [57] stated that in vitro induced genetic rearrangements in *Oryza sativa* could have taken place in not coding areas of the genome so they did not affect phenotype. That could also be the case for *S. myrtilloides*. There are examples of plants regenerated from organized meristems, which anyway present some participation of PCR polymorphic products [20,21,58,59], but without phenotypic variation. When a related *S. lapponum* species was regenerated by Parzymies et al. [52], some polymorphic bands were also amplified. It can be hypothesized that susceptibility to minor genetic rearrangements is a species/genotype-dependent feature, as Devarumath et al. [60] also stated in the case of three tea clones.

### 3.5. Flow Cytometry

**Figure 5:**
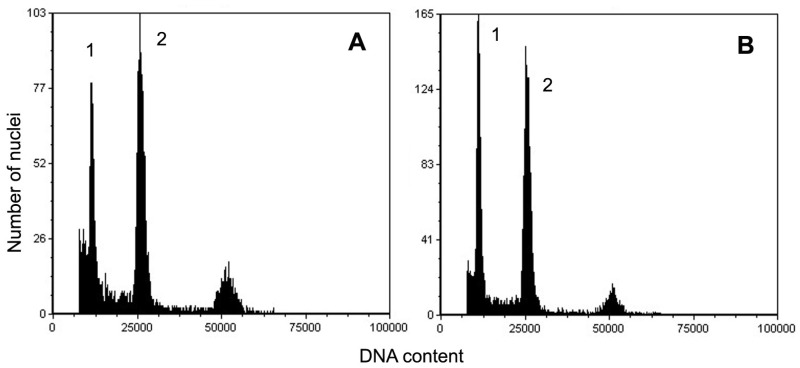
Flow cytometric histograms of nuclear DNA content of *Salix myrtilloides*. (**A**) plant from natural habitat; (**B**) plantlet produced in vitro. 1—the G_0_/G_1_ peak of *S. myrtilloides*; 2—the G_0_/G_1_ peak of an internal standard (*Solanum lycopersicum*).

The nuclear DNA content of *S. myrtilloides* was 0.887 (±0.0102) pg/2C (mean ± SD) in the leaves of control plants collected from the natural habitat and 0.847 (±0.0173) pg/2C (mean ± SD) in plantlets produced in vitro (Figure 5).

Those values did not differ significantly (*p* = 0.05), thus flow cytometric analysis confirmed the genome size stability of micropropagated material, and the usefulness of developed protocol for the production of plant material of this species in vitro. Additionally, in our previous report [22], where we developed micropropagation protocol for *S. lapponum*, no somaclonal variation occurred in the plantlets. Stable plant material was produced in vitro for many other species, including trees and shrubs [52,61,62,63,64,65]. Such stability is a prerequisite for practical application of such material.

To the best of our knowledge, this is the first report of the genome size of *S. myrtilloides*. The value established (about 0.85 pg/2C) was similar to that obtained for *S. lapponum* (0.87 pg/2C), and it also falls within the range of 2C-values of different diploid *Salix* species included in the Kew Plant DNA C-values Database, which varies between 0.70 pg/2C and 0.96 pg/2C [66].

## 4. Conclusions

Different studies conducted so far suggest that in vitro cultures of endangered species can be important in active conservation procedures. Micropropagation allows the preservation of genetic resources and production of plants that can be later used in different ways.

In the case of *S. myrtilloides*, the obtained regenerants may be used to strengthen the existing population and establish new ones. The tissue culture protocol presented in the manuscript may be applied in the propagation of the species studied and other willow species. The results of the conducted genetic analyses are also given, which show that the cultivation in vitro provides true-to-type regenerated plants, which can be used for establishing future populations.

## Figures and Tables

**Table 1:** Composition and concentrations of reagents used during ISSR analysis.

Reagent	Concentration
PCR buffer (750 mM Tris pH 8.8; 200 mM (NH_4_)_2_SO_4_; 0.1% Tween 20)	1×
MgCl_2_	3 mM
dNTP	200 μM (each)
Primer	670 nM
*Taq* DNA Polymerase (Thermo Fisher Scientific Waltham, MA, USA)	0.5 U

**Table 2:** Thermocycling program used for ISSR analysis.

	Initial Denaturation	Touchdown Cycles			Enrichment Cycles			Final Extension
		Denaturation	Annealing	Elongation	Denaturation	Annealing	Elongation	
Temperature (°C)	94	94	54→52 (−1 °C each 3 cycles)	72	94	52	72	72
Time (s)	120	30	45	120	30	45	120	7
Number of cycles	-	6			32			-

**Table 3:** Disinfection efficiency and regeneration rate of *S. myrtilloides* explants depending on the disinfection method.

Disinfection Method	Number of Regenerating Explants without Contamination n/N (%)	Number of Necrotic Explants nec/N (%)
NaOCl 1.5%	66/170 (38.8%)	44/170 (25.9%)
AgNO_3_ 0.5%	23/164 (14.0%)	118/164 (72.0%)
Two-step disinfection	24/140 (17.1%)	53/140 (37.9%)

n—number of uncontaminated regenerating shoots, N—number of explants used, nec—number of necrotic explants.

**Table 4:** Regeneration and multiplication of *S. mytilloides* shoots in tissue culture depending on the cytokinins content in the media.

Cytokinin	Concentration (mg·dm^−3^)	Main Shoot Length (mm)	Number of Nodes	Main Shoot Weight (mg)	Plants with Axillary Shoots (%)	Number of Axillary Shoots/Plant	Mn Rate **
K	0	40.24 bd *	11.26 a	68.87 ac	5	3.0 ab	5.78
BA	0.1	17.71 df	6.61 bc	68.48 ac	67	1.67 b	4.43
	0.5	61.00 ab	10.90 a	108.81 a	65	2.08 b	6.80
	1	38.00 ce	8.50 ab	69.53 ac	90	1.94 b	6.00
	2.5	23.65 df	6.35 ad	45.73 ac	41	2.57 b	4.23
KIN	0.1	47.90 ac	7.60 b	94.86 ab	25	1.20 b	4.10
	0.5	67.26 a	9.21 ab	124.00 a	16	1.00 b	4.77
	1	68.05 a	8.42 ab	138.66 a	5	1.00 b	4.26
	2.5	58.80 ab	7.70 b	100.47 ab	25	1.40 b	4.20
2iP	0.1	24.78 cf	7.11 b	62.27 ac	79	4.87 a	7.41
	0.5	12.59 ef	7.75 b	56.02 ac	100	2.89 b	6.77
	1	38.38 ce	3.53 d	30.42 bc	69	2.44 b	3.56
	2.5	11.00 f	3.75 cd	23.26 c	55	1.18 b	2.42

* values in columns with the same letter do not differ significantly at *p* = 0.05; ** Mn rate—multiplication rate.

**Table 5:** Rooting of *S. myrtilloides* shoots in tissue culture depending on cytokinins added to the media.

Cytokinin	Concentration (mg·dm^−3^)	Number of Rooted Shoots (%)	Number of Roots	Length of Roots (mm)	Weight of Roots (mg)	Number of Shoots with Callus (%)	Weight of Callus (mg)
K	0	100	4.11 ab *	22.75 b	2.01 d	0	-
BA	0.1	5	2.00 b	23.00 ab	1.85 d	33	20.37 ab
	0.5	100	4.65 ab	42.03 a	13.23 bc	50	24.43 ab
	1	100	5.45 a	52.00 a	11.97 bc	80	23.85 ab
	2.5	95	5.13 a	42.66 a	11.24 bc	76	41.06 a
KIN	0.1	100	3.30 ab	41.15 a	10.30 bc	15	1.70 b
	0.5	100	3.21 ab	41.08 a	13.74 bc	0	-
	1	95	3.00 ab	47.56 a	21.72 ab	0	-
	2.5	95	2.94 ab	46.71 a	28.97 a	0	-
2iP	0.1	68	5.31 a	42.18 a	6.71 c	83	35.33 ab
	0.5	0	-	-	-	100	33.22 ab
	1	0	-	-	-	72	38.54 ab
	2.5	0	-	-	-	0	-

* values in columns with the same letter do not differ significantly at *p* = 0.05.

**Table 6:** ISSR primers used in *S. myrtilloides* analysis.

Primer	Sequence	Products Size Range (bp)
SR1	(AG)_8_G	350–1150
SR14	(GA)_8_YG	300–1500
SR16	(GA)_8_C	320–950
SR32	(AG)_8_YT	200–620
SR75	(AT)_8_C	640–1600

Y-T or C; R = G or A.

## Data Availability

All the required data, which are related to the current study, are embedded in this manuscript.
